# The differences of bacterial communities in the tissues between healthy and diseased Yesso scallop (*Patinopecten yessoensis*)

**DOI:** 10.1186/s13568-019-0870-x

**Published:** 2019-09-14

**Authors:** Zichao Yu, Chao Liu, Qiang Fu, Guangxia Lu, Shuo Han, Lingling Wang, Linsheng Song

**Affiliations:** 1 Liaoning Key Laboratory of Marine Animal Immunology and Disease Control, Dalian Ocean University, Dalian, 116023 China; 2 Functional Laboratory of Marine Fisheries Science and Food Production Processes, Qingdao National Laboratory for Marine Science and Technology, Qingdao, 266235 China; 3 Liaoning Key Laboratory of Marine Animal Immunology, Dalian Ocean University, Dalian, 116023 China; 4 Dalian Key Laboratory of Prevention and Control of Aquatic Animal Diseases, Dalian Ocean University, Dalian, 116023 China

**Keywords:** Yesso scallop, Bacterial community, 16S rRNA, Functional composition

## Abstract

The tissues of marine invertebrates are colonized by species-rich microbial communities. The dysbiosis of host’s microbiota is tightly associated with the invertebrate diseases. Yesso scallop (*Patinopecten yessoensis*), one of the most important maricultured scallops in northern China, has recently suffered massive summer mortalities, which causes huge production losses. The knowledge about the interactions between the Yesso scallop and its microbiota is important to develop the strategy for the disease prevention and control. In the present study, the bacterial communities in hemolymph, intestine, mantle and adductor muscle were compared between the healthy and diseased Yesso scallop based on the high-throughput sequencing of 16S rRNA gene. The results indicated obvious difference of the composition rather than the diversity of the bacterial communities between the healthy and diseased Yesso scallop. *Vibrio*, *Francisella* and *Photobacterium* were found to overgrow and dominate in the mantle, adductor muscle and intestine of the diseased scallops, respectively. The prediction of bacterial community metagenomes and the variations of KEGG pathways revealed that the proportions of the pathways related with neurodegenerative diseases and carbohydrate metabolism both increased significantly in the mantle and hemolymph of the diseased scallops. The abundance of the metabolism pathways including carbohydrate metabolism, lipid metabolism and amino acid metabolism decreased significantly in the intestine of diseased scallops. The results suggested that the changes of bacterial communities might be closely associated with the Yesso scallop’s disease, which was helpful for further investigation of the pathogenesis as well as prevention and control of the disease in Yesso scallop.

## Introduction

Microbiota has been believed to play vital roles in the survival, homeostasis and development of vertebrate and invertebrate hosts (McFall-Ngai et al. 2013; Thaiss et al. 2016; Wang and Jia 2016). Accumulating studies have been focused on the interactions between the microbiota and its host to provide valuable information for clinical diagnoses, therapy as well as further investigation of the pathogenesis (De Schryver and Vadstein 2014; Rooks and Garrett 2016; Thaiss et al. 2016). A large number and diversity of microbial communities usually colonize in tissues such as hemolymph and intestine of the healthy marine invertebrates (Lokmer and Wegner 2015; Lu et al. 2017; Meisterhans et al. 2015), which are of pivotal importance for preventing the outcome of pathogenic bacteria (Defer et al. 2013), modulating the host’s immune system (Schmitt et al. 2012), and promoting nutrient absorption (Yamazaki et al. 2016). It has been broadly recognized that the disturbance of the commensal microbial communities resulting from environmental stress can cause disease of invertebrate. The dysbiosis in the symbiotic microbiota was found to precede the first symptoms of the invertebrate hosts (Dubert et al. 2016; Xiong 2018), suggesting it was the first responder to perturbation and could accelerate the disease progression (Xiong et al. 2015). The hemolymph microbiome of Pacific oysters could be significantly affected by temperature or temperature stress, and the decreased diversity of the bacterial communities might contribute to oyster’s high mortality rates (Lokmer and Wegner 2015). The degree of the intestinal flora alteration in shrimp was also found to be closely linked with the disease severity (Chen et al. 2017; Xiong 2018). Therefore, characterization of the microbiota variation between healthy and diseased hosts is helpful to investigate the pathogenesis and develop therapy for the microbial diseases in marine invertebrates.

In the past decade, shellfish aquaculture has been greatly plagued by repeated episodes of bacterial diseases all over the world (Cheikh et al. 2016; Romalde et al. 2014). Many maricultured species suffered from the frequent outbreaks of the massive mortalities, such as oysters, scallops, clams and mussels (Garnier et al. 2007; Liu et al. 2013; Travers et al. 2015), which severely reduced the production and caused high economic losses. The mechanisms of the occurrence, development and outcome of shellfish diseases are complicated, which involve the pathogenic microbes, shellfish, and aquaculture environment. Although vibrios have been described as the main pathogens for shellfish diseases (Romalde et al. 2014), it’s difficult to provide a comprehensive insight into the bacterial species associated with the shellfish diseases. Co-infection of multiple pathogens in oyster has been found to enhance their virulence under field conditions (Saulnier et al. 2010), and the discovery have questioned the traditional Koch’s postulates about a pathogen causing a disease, or a virulence gene causing a disease (Falkow 1988). Moreover, the diseases of aquatic animals are considered as the consequence of interactions among the hosts, internal microbiota and ambient environment (De Schryver and Vadstein 2014; Xiong 2018), and the internal microbiota is regarded as the bridge between the hosts and the surrounding environment (De Schryver and Vadstein 2014; Wang 2018). Hence, the investigation of microbiota shift between healthy and diseased shellfish is helpful for identification of potential polymicrobial pathogens and understanding their pathogenesis in shellfish.

Yesso scallop *Patinopecten yessoensis* is a cold water bivalve, which is naturally distributed in northern Japan, northern Korean Peninsula and Far East of Russian (Hou et al. 2011). It has become one of the most important maricultured shellfishes in northern China since it was introduced into China in the 1980s (Wang 1984). After having thrived for many years, the development of Yesso scallop aquaculture has been largely hampered by frequent outbreaks of massive mortalities since 2009 (Liu et al. 2013; Teng et al. 2012), which leads to great loss of up to about 200 million RMB to the local economy every year. In spite of substantial efforts to detect and isolate the pathogens (Itoh et al. 2013; Liu et al. 2013, 2016; Meyer et al. 2017; Teng et al. 2012), the knowledge about the relationship between disease occurrence and internal microbiota of Yesso scallop is still very limited, which makes it the bottle-neck to further study the pathogenesis of Yesso scallop’s disease. In the present study, the diversities of bacterial community in hemolymph, intestine, mantle and adductor muscle of healthy and diseased Yesso scallops were investigated by high-throughput sequencing of V4 region of 16S rRNA gene. The objectives of this study were to compare the compositions of bacterial communities in different tissues between healthy and diseased Yesso scallop, and assess the potential roles of bacterial communities in the diseased Yesso scallop.

## Materials and methods

### Sample collection

Seawater and Yesso scallops were collected from a commercial scallop farm (39°16′53.0″N, 122°44′09.0″E) located in Dalian, Liaoning Province, China in July 2017. Seawater was collected at the depth of 3 m where the scallops were suspension-fed, and stored in polyethylene flasks. The seawater samples were transported to the laboratory at 4 °C within an hour before the filtration. Scallops with an average shell length of 66 ± 4 mm and average weight of 34 ± 6 g were collected from the suspended cage. The diseased scallops were characterized by the atrophy of mantle and abscess on adductor muscle, and the morbidity was 22.5%. The scallops with no lesions were selected as the healthy group. The scallop shell surface was first washed with sterile seawater and disinfected with 70% ethanol. After the shells were opened, the scallops were washed with sterile seawater to remove the residual seawater. Samples of hemolymph (HE), intestine (IN), mantle (MA) and adductor muscle (MU) were collected from the healthy (H) and diseased (D) scallops, respectively. The tissues from three individuals were pooled together, and there were three replicates for each type of tissue. All of the samples were stored at − 80 °C before DNA extraction.

### Genomic DNA extraction and high-throughput sequencing

Seawater samples (2 L/replicate × 3 replicates) were filtered by 0.22 μm pore size membrane (Sagon Biotech, Shanghai, China) to enrich the microbial cells. The membrane was cut into four pieces, and total genomic DNA was extracted with the Water DNA Kit (Omega, Norcross, GA, USA) following the manufacturer’s instruction. Intestine, mantel and adductor muscle samples were first homogenized, and hemolymph samples were centrifuged at 13,000 rpm for 10 min to acquire the precipitate. The total genomic DNA of the samples was then extracted with the Soil DNA Kit (Omega, Norcross, GA, USA) following the manufacturer’s instructions. DNA quality and quantity were checked by 1% agarose gel electrophoresis and NanoDrop spectrophotometer (Thermo Fisher Scientific, Inc, Wilmington, DE, USA). The V4 region of 16S rRNA gene was amplified using the primers of 515F (GTGBCAGCMGCCGCGGTAA) and 806R (GGACTACHVGGGTWTCTAAT). The high-throughput sequencing of V4 region of 16S rRNA gene was accomplished on the Illumina PE250 platform (Novogene, Beijing, China), along with quality filter (removal of singletons and chimeric sequences) of raw sequence reads. The raw sequence data reported in this paper have been deposited in the Genome Sequence Archive (Wang et al. 2017) in BIG Data Center (Zhang et al. 2019), Beijing Institute of Genomics (BIG), Chinese Academy of Sciences, under accession numbers CRA001744 that are publicly accessible at http://bigd.big.ac.cn/gsa.

### Diversity analysis and function prediction of the bacterial community

The software package of Quantitative Insights Into Microbial Ecology (QIIME) (Caporaso et al. 2010) was used to estimate α- and β-diversity of the bacterial communities. Operational Taxonomic Unit (OTU) table was first created with uclust (Edgar 2010) against Silva database version 128 (Quast et al. 2012) at a 97% similarity threshold. The OTUs affiliated with Archaea and Chloroplast as well as those less than 0.1% were filtered out from the OTU table. The α-diversity metrics including observed OTUs, Chao1 and Shannon diversity index, and the composition of the bacterial communities as well as the rarefaction curves were calculated and visualized. In β-diversity analysis, the sequence reads of all the samples were normalized to the minimum count to avoid the basis caused by different sequence depth, and principal coordinates analysis (PCoA) plot based on the weighted UniFrac metrics was used to visualize the dissimilarity in bacterial community compositions of different samples. Phylogenetic Investigation of Communities by Reconstruction of Unobserved States (PICRUSt2; https://github.com/picrust/picrust2/) was used for predicting functional abundances of the bacterial communities based on the OTU table generated by QIIME and the representative sequence data from Silva database version 128. The prediction of KO abundances was carried out with Hidden-state prediction (Zaneveld and Thurber 2014), and then the KOs were collapsed into pathways.

### Real-time quantitative PCR

Total bacterial number in different samples was assessed by absolute quantitative PCR according to previous study (Lu et al. 2017). In brief, the fragment of 16S rRNA gene was amplified from the genomic DNA of *Vibrio splendidus* JZ6 (Liu et al. 2013) with primers 341F/534R (Bru et al. 2008) using Ex Taq DNA polymerase (TaKaRa, Dalian, China), and the amplicon was inserted into pMD-18T vector (TaKaRa, Dalian, China) to construct the standard plasmid. SYBR Green fluorescent RT-qPCR was performed on ABI PRISM 7500 Sequence Detection System (Applied Biosystems, Foster City, CA, USA) using gradiently diluted standard plasmid templates and primers 341F/534R. The standard curve was constructed with the threshold cycle (Ct) values against the denary logarithms of the target gene’s copy numbers. For the estimation of the total bacterial number, total genomic DNA of different samples was adjusted to 10 ng/μL. After RT-qPCR with primers 341F/534R, the Ct values were recorded, and the 16S rRNA gene copy numbers in different samples were calculated based on the standard curve.

### Statistical analysis

Linear discriminant analysis (LDA) effect size (LEfSe) (Segata et al. 2011) was employed to identify the significantly differentially abundant taxa between the tissues of healthy and diseased scallops based on the top 100 most abundant species. The alpha value for the factorial Kruskal–Wallis test was set to 0.05, and threshold on the logarithmic LDA score for discriminative features was set to 3.5. The results were visualized in the form of taxonomic cladogram. Statistical Package for Social Sciences (SPSS) 19.0 (SPSS INC, Chicago, IL, USA) was employed for the statistical analysis of the data obtained. The quantitative data were expressed as mean ± SD. Significant differences among multiple groups were tested with One-way analysis of variance (ANOVA), followed by Turkey’s post hoc test. One asterisk (*) indicated statistical significance (*p* < 0.05), and double asterisks (**) indicated extremely statistical significance (*p* < 0.01).

## Results

### Characteristics of the high-throughput sequence data

High-throughput sequencing of 16S rRNA gene V4 region was conducted to analyze the diversity of bacterial communities in different tissues of the healthy and diseased scallops. A total of 1,084,329 reads (Additional file 1: Table S1) were obtained after data quality filter processing. The effective reads were clustered into 545 OTUs affiliated with Bacteria. The rarefaction curve of each individual sample was almost saturated (Additional file 2: Fig. S1), and the Good’s coverage indexes of all of the samples were more than 0.99 (data not shown), indicating that the sequencing depth was adequate.

### The diversity of bacterial communities and bacterial loads

**Fig. 1:**
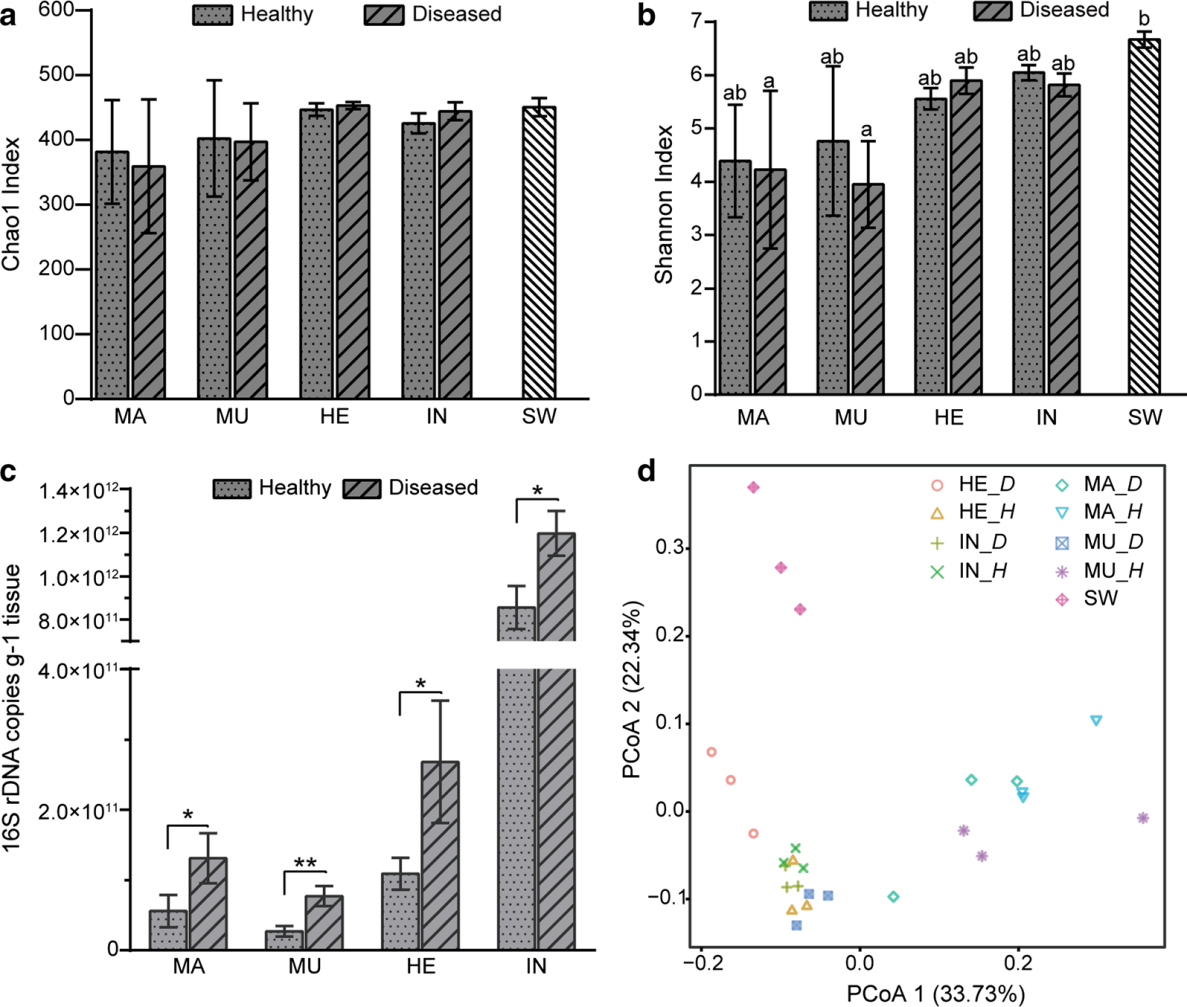
Bacterial diversity comparisons in different tissues between healthy and diseased Yesso scallop. **a** Chao1 index of different samples. **b** Shannon index of different samples. **c** Copy number of 16S rRNA gene in different samples. **d** PCoA plot of the bacterial communities in different samples based on the weighted UniFrac metrics. MA, mantle; MU, adductor muscle; HE, hemolymph; IN, intestine; SW, seawater; *H*, healthy; *D*, diseased. Lowercase letters above the error bars indicate statistical significance among samples

The complexity of bacterial communities in seawater and different tissues of the healthy and diseased scallops was evaluated based on the α-diversity analysis. The bacterial community in seawater exhibited a relatively higher Chao1 index and the highest Shannon index compared with the tissue samples (Fig. 1a, b). The Chao1 and Shannon index were higher in hemolymph and intestine among the tissue samples irrespective of the host’s health status (Fig. 1a, b). Variations of the α-diversity were observed in the tissues between the healthy and diseased Yesso scallop, but there were no significant differences. There was obvious differences in the bacterial abundance and community structure. In the healthy scallop, the bacteria number was the highest in intestine and lowest in adductor muscle. The bacterial loads were significantly higher in all of the detected tissues of diseased scallop, compared to those in the healthy scallop (Fig. 1c). The 16S rRNA gene copy number per gram tissue was 5.58 × 10^10^ and 1.31 × 10^11^ in the mantles, 2.70 × 10^10^ and 7.71 × 10^10^ in the adductor muscles, 1.09 × 10^11^ and 2.68 × 10^11^ in the hemolymph, 8.56 × 10^11^ and 1.20 × 10^12^ in the intestines of healthy and diseased scallop, respectively. PCoA analysis was conducted to illustrate the dissimilarity of the bacterial community structure in different samples (Fig. 1d). The bacterial communities in the scallop were remarkably distinct from that in the surrounding seawater. Moreover, the samples of MU_H and MU_D formed two clusters that distinctly separated from each other. Similarly, the samples of HE_H and HE_D also formed two distinct clusters, but the distance between them was closer than that between the MU_H and MU_D samples. Even there was some overlaps, the distinct patterns were observed between the IN_D and IN_H samples, as well as the MA_D and MA_H samples.

### The differences of bacterial community compositions in mantle

**Fig. 2:**
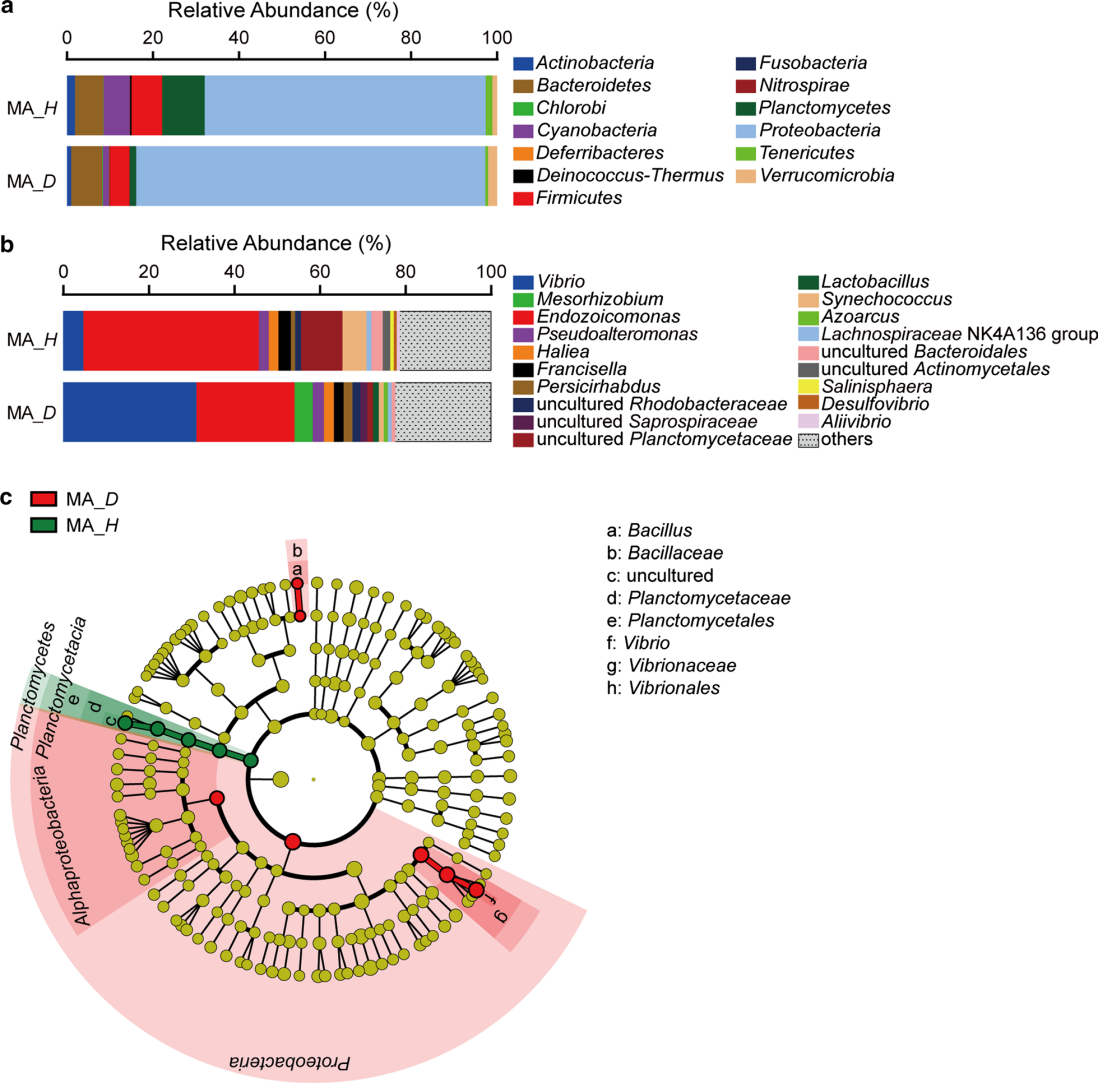
Composition of the bacterial communities in the mantle of healthy and diseased scallops. **a** Composition of the bacterial communities at phylum level. **b** Composition of the bacterial communities at genus level. The top 15 abundant genera were shown in the figure and the rest was indicated as “Others”. **c** The most differentially abundant taxa between the mantle of healthy and diseased scallops based on the taxonomic cladogram obtained from LEfSe. MA, mantle; *H*, healthy; *D*, diseased

There were 13 major phyla in the bacterial communities in the mantle of healthy and diseased scallops (Fig. 2a). In the mantle of healthy scallop, *Proteobacteria* (65.35%) was the most dominant phylum. *Bacteroidetes* (6.63%), *Cyanobacteria* (6.04%), *Firmicutes* (7.09%) and *Planctomycetes* (9.85%) also exhibited relatively higher abundance. In the mantle of diseased scallop, *Proteobacteria* (81.21%) was the most abundant phylum, and the abundance of *Bacteroidetes* (7.19%) and *Firmicutes* (4.50%) was relatively higher. The abundance of *Proteobacteria* in the mantle of diseased scallop was higher than that in the mantle of healthy scallop. While the abundances of *Cyanobacteria*, *Firmicutes* and *Planctomycetes* in the mantle of healthy scallop were higher than that in the diseased scallop.

At the genus level, the proportion of *Endozoicomonas* (50.00%) was the highest in the mantle of healthy scallop, followed by a group of uncultured *Planctomycetaceae* (9.63%) and *Synechococcus* (5.63%) (Fig. 2b). In the mantle of diseased scallop, the abundance of *Vibrio* was 31.00%, which was much higher than that in the mantle of healthy scallop (4.62%). *Endozoicomonas* was the second highest abundant genus (22.96%) in the diseased group, which was lower than that in the healthy group. LEfSe analysis was performed to find the differentially distributed bacteria between the healthy and diseased scallop (Fig. 2c). *Vibrio* and *Bacillus* were found to be significantly enriched in the diseased scallop, although the abundance of *Bacillus* was low in mantle. However, a group of uncultured *Planctomycetaceae* belonging to *Planctomycetes* was obviously enriched in healthy scallop.

### The differences of bacterial community compositions in adductor muscle

**Fig. 3:**
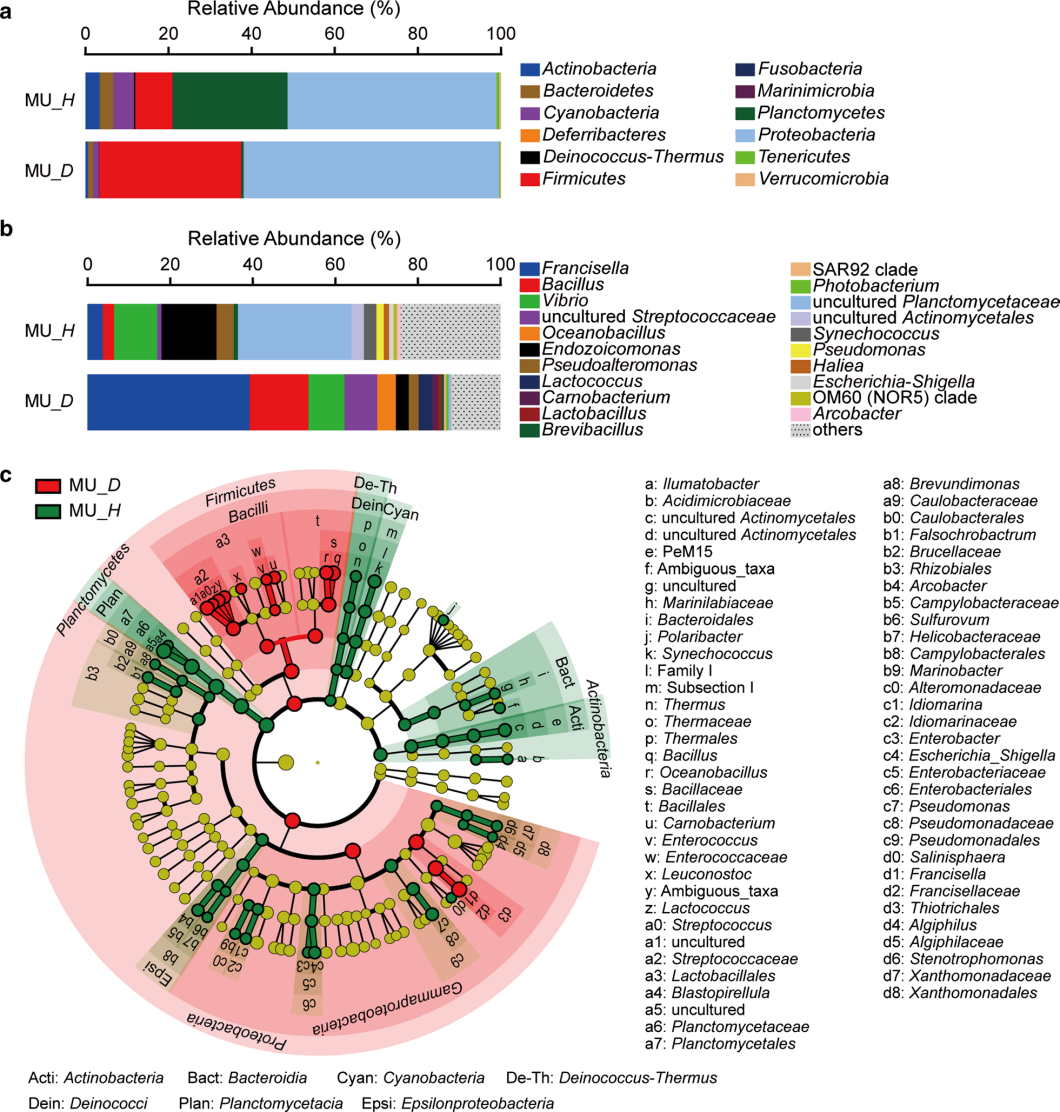
Composition of the bacterial communities in the adductor muscle of healthy and diseased scallops. **a** Composition of the bacterial communities at phylum level. **b** Composition of the bacterial communities at genus level. The top 15 abundant genera were shown in the figure and the rest was indicated as “Others”. **c** The most differentially abundant taxa between the adductor muscle of healthy and diseased scallops based on the taxonomic cladogram obtained from LEfSe. MU, adductor muscle; *H*, healthy; *D*, diseased

There were 12 major phyla in the bacterial communities of adductor muscle (Fig. 3a). The top three most abundant phyla were *Proteobacteria*, *Planctomycetes* and *Firmicutes* in the adductor muscle of healthy scallop, and their proportions were 50.24%, 27.63% and 9.02%, respectively. In the diseased samples, *Proteobacteria* (61.50%) and *Firmicutes* (34.16%) dominated in the bacterial community, and their abundances were higher than that in the healthy samples. Nevertheless, the abundances of *Planctomycetes*, *Actinobacteria*, *Bacteroidetes* and *Cyanobacteria* were far lower in the diseased samples (0.56% to 1.37%).

At the genus level, the bacterial community was dominated by a group of uncultured *Planctomycetaceae* (27.43%), *Endozoicomonas* (13.34%) and *Vibrio* (10.46%) in the healthy scallop (Fig. 3b). *Francisella* (39.16%), *Bacillus* (14.25%), *Vibrio* (8.75%) and a group of uncultured *Streptococcaceae* (7.91%) dominated in the bacterial community in the adductor muscle of diseased scallop. Ten genera of bacteria were found to significantly overgrow in the adductor muscle of diseased scallop (Fig. 3c), including *Francisella*, *Bacillus*, *Oceanobacillus*, *Carnobacterium*, *Lactobacillus*, uncultured *Streptococcaceae*, *Enterococcus*, *Leuconostoc,* and *Streptococcus*. Except *Francisella* that was affiliated with *Proteobacteria*, the other overgrowing bacteria were all affiliated with *Firmicutes*. In addition, 21 genera of bacteria were significantly enriched in the adductor muscle of healthy scallop, which were affiliated with *Planctomycetaceae*, *Deinococcus*-*Thermus*, *Cyanobacteria*, *Bacteroidetes*, *Actinobacteria*, and *Proteobacteria*.

### The differences of bacterial community compositions in hemolymph

**Fig. 4:**
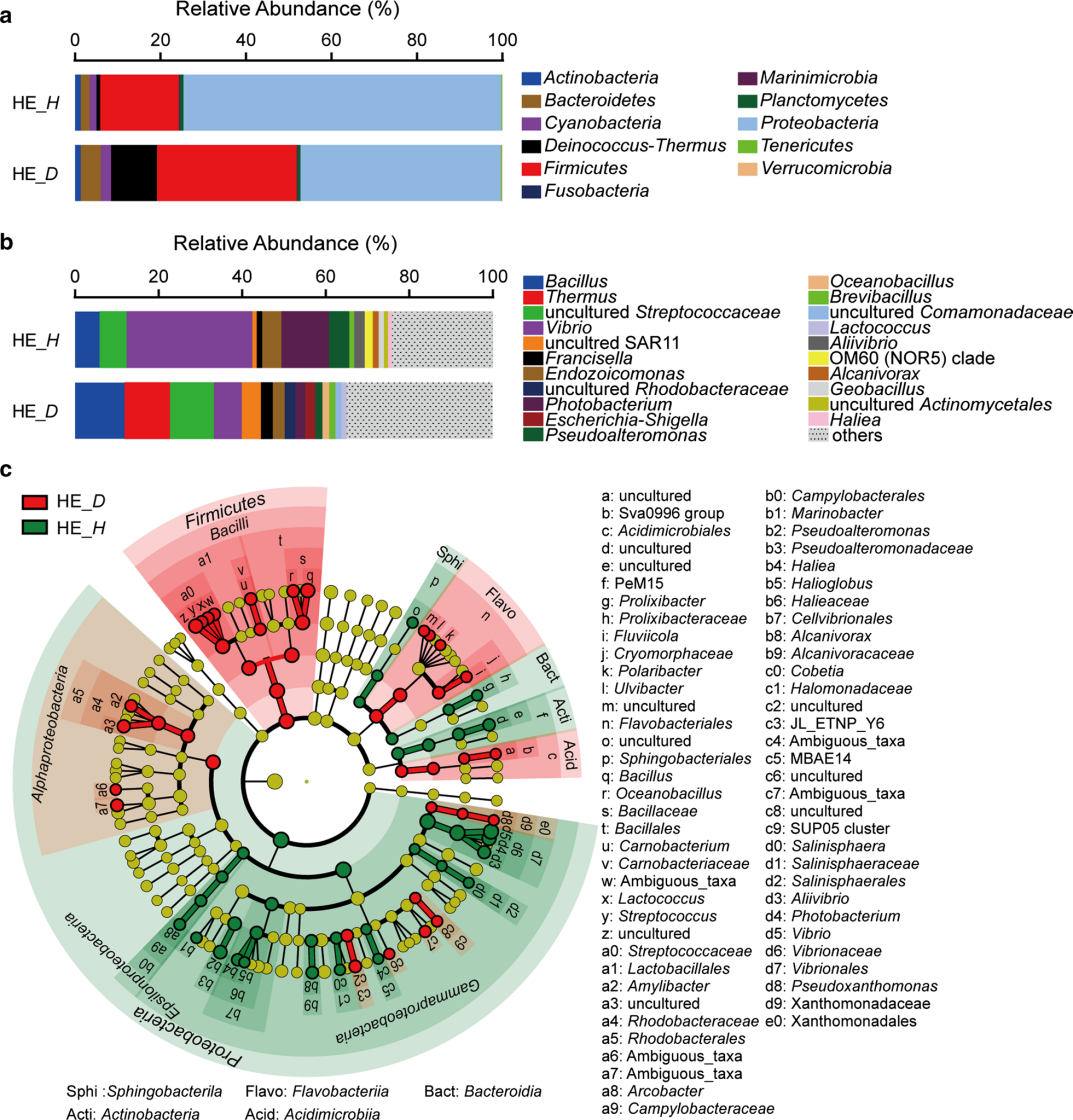
Composition of the bacterial communities in the hemolymph of healthy and diseased scallops. **a** Composition of the bacterial communities at phylum level. **b** Composition of the bacterial communities at genus level. The top 15 abundant genera were shown in the figure and the rest was indicated as “Others”. **c** The most differentially abundant taxa between the hemolymph of healthy and diseased scallops based on the taxonomic cladogram obtained from LEfSe. HE, hemolymph; *H*, healthy; *D*, diseased

There were 11 major phyla in the bacterial communities of hemolymph (Fig. 4a). In the hemolymph of healthy scallop, *Proteobacteria* showed the absolute predominance (74.30%), and *Firmicutes* (18.38%) was the second most abundant phylum, which together made up more than 90% of the bacterial population. In the hemolymph of diseased scallop, *Proteobacteria* was the most abundant phylum (46.90%), followed by *Firmicutes* (32.65%) and *Deinococcus*–*Thermus* (10.78%) whose abundance was less than 1% in the hemolymph of healthy scallop.

At the genus level, *Vibrio* was the most abundant bacteria in the hemolymph of healthy scallop, which accounted for 30.10% of the whole population (Fig. 4b). *Photobacterium* was the second most abundant bacteria, making up 11.42% of the bacterial community in the hemolymph of healthy scallop. Both of their abundances were higher than those in the hemolymph of diseased scallop. The proportions of *Bacillus* (11.84%), *Thermus* (10.78%), a group of uncultured *Streptococcaceae* (10.53%), *Vibrio* (6.67%) and a group of uncultured SAR11 (4.58%) were relatively high in the hemolymph of diseased scallop. Based on LEfSe analysis (Fig. 4c), 21 genera were found to be obviously enriched in the hemolymph of diseased scallop, which were distributed in the classes of *Bacilli* (7 genera, such as *Bacillus*, *Oceanbacillus* and *Lactococcus*), *Flavobacteriia* (4 genera, such as *Fluviicola* and *Polaribacter*), *Alphaproteobacteria* (4 genera, such as *Amylibacter*), *Gammaproteobacteria* (5 genera, such as *Pseudoxanthomonas*), and *Acidimicrobiia* (1 genera). And 15 genera were found to significantly overgrow in the hemolymph of healthy scallop, which were distributed in the classes of *Gammaproteobacteria* (12 genera, such as *Vibrio*, *Photobacterium* and *Pseudoalteromonas*), *Actinobacteria* (uncultured PeM15 clade), *Bacteroidia* (*Prolixibacter*), *Sphingobacterila* (uncultured *Sphingobacteriales*), and *Epsilonproteobacteria* (*Arcobacter*).

### The differences of bacterial community compositions in intestine

**Fig. 5:**
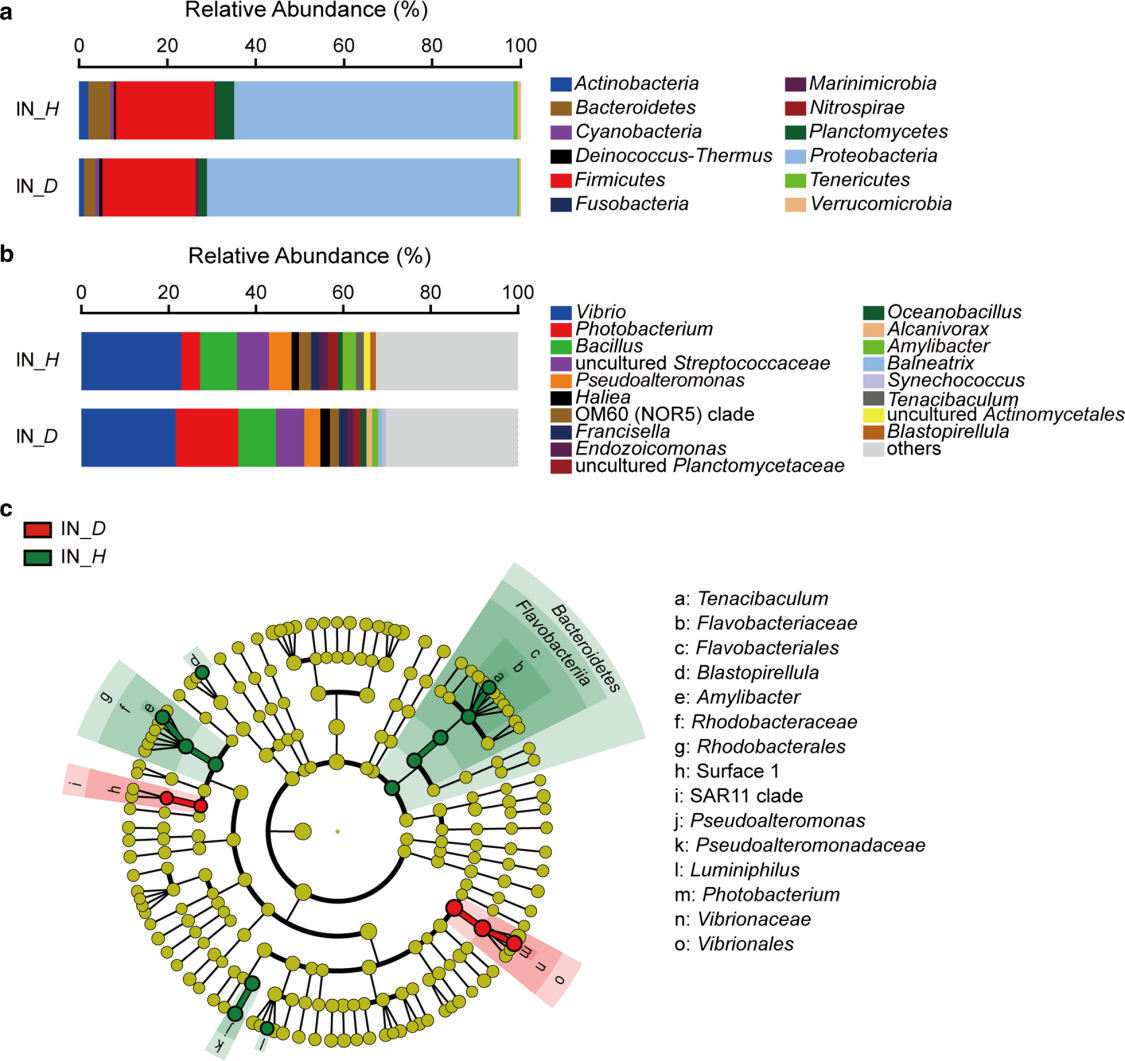
Composition of the bacterial communities in the intestine of healthy and diseased scallops. **a** Composition of the bacterial communities at phylum level. **b** Composition of the bacterial communities at genus level. The top 15 abundant genera were shown in the figure and the rest was indicated as “Others”. **c** The most differentially abundant taxa between the intestine of healthy and diseased scallops based on the taxonomic cladogram obtained from LEfSe. IN, intestine; *H*, healthy; *D*, diseased

The bacterial OTUs were clustered into 12 phyla in the intestine samples (Fig. 5a). The top four most abundant phyla were *Proteobacteria*, *Firmicutes*, *Bacteroidetes* and *Planctomycetes* in both healthy and diseased scallops. Their abundances were 63.30% and 70.22% for *Proteobacteria*, 22.35% and 21.09% for *Firmicutes*, 4.93% and 2.54% for *Bacteroidetes*, 4.14% and 1.92% for *Planctomycetes* in healthy and diseased scallop, respectively.

*Vibrio* was the predominant genus in the intestine of both healthy and diseased scallops (Fig. 5b), with abundances of more than 20%. *Bacillus*, a group of uncultured *Streptococcaceae*, *Photobacterium* and *Pseudoalteromonas* were also abundant in both healthy and diseased scallops. LEfSe analysis revealed that *Photobacterium* and Surface 1 of SAR11 clade were significantly enriched in the intestine of diseased scallop (Fig. 5c), which were 14.37% vs. 4.34% for *Photobacterium* and 0.50% vs. 0.06% for Surface 1 of SAR11 clade in the diseased and healthy scallop, respectively. *Tenacibaculum* (1.80% vs. 0.38%), *Blastopirellula* (1.29% vs. 0.25%), *Amylibacter* (3.02% vs. 1.33%), *Pseudoalteromonas* (5.20% vs. 3.69%) and *Luminiphilus* (0.84% vs. 0.22%) were significantly enriched in the intestine of healthy scallop.

### The functional variations of bacterial communities

**Fig. 6:**
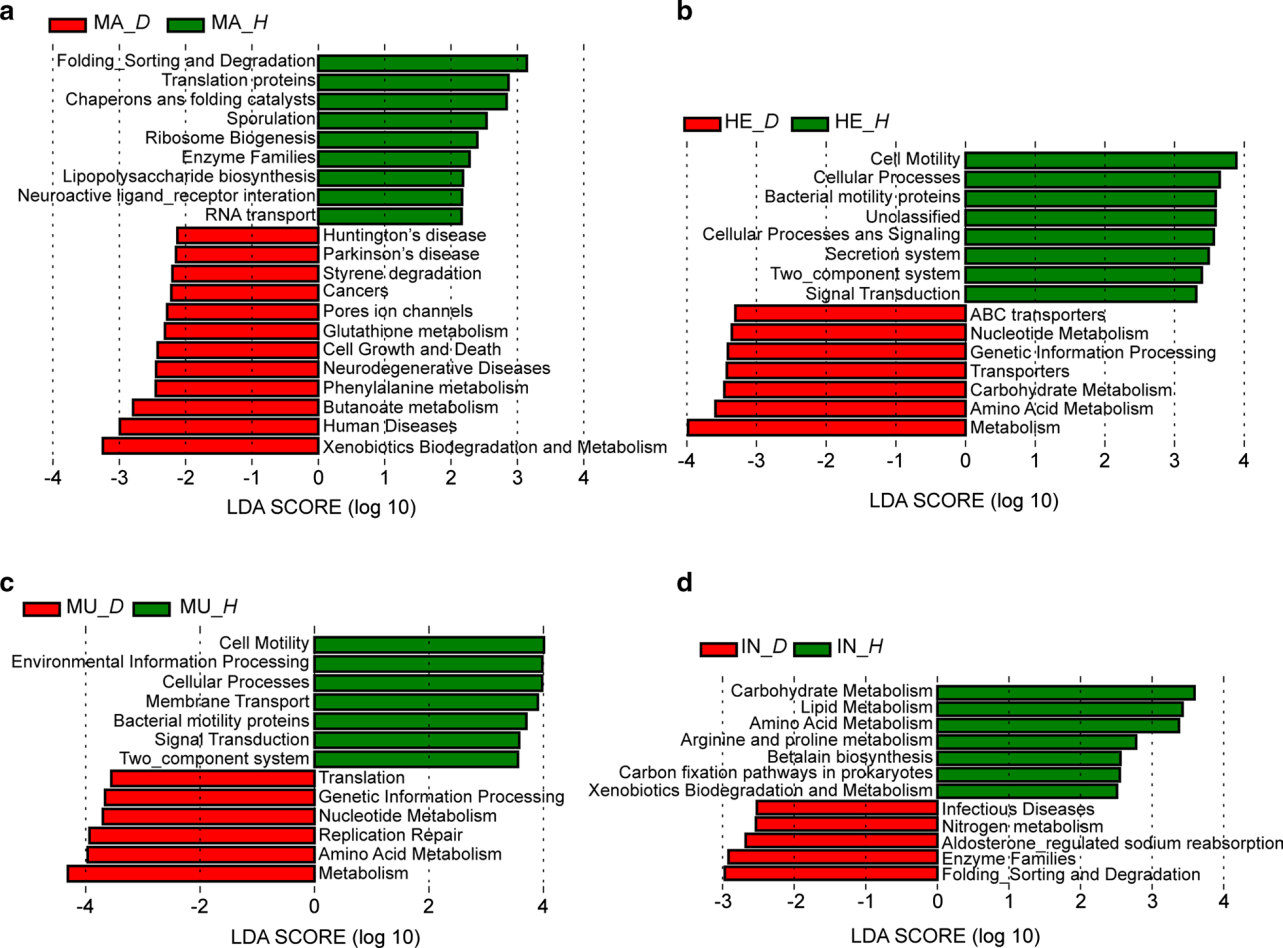
The most differentially abundant predicted function of the bacterial communities in mantle (**a**), hemolymph (**b**), adductor muscle (**c**) and intestine (**d**) between the healthy and diseased Yesso scallop identified by LEfSe. *H*, healthy; *D*, diseased

The functions of the bacterial communities in healthy and diseased Yesso scallops were predicted by PICRUSt program based on KEGG pathways. The LEfSe was used to explore the differences of the bacterial functions between the healthy and diseased scallop. The pathways associated with human diseases and metabolism varied significantly between healthy and diseased scallops. The pathways related to neurodegenerative diseases including Huntington’s disease and Parkinson’s disease were significantly enriched in the mantle of diseased scallop (Fig. 6a). The abundances of the pathways involved in nucleotide metabolism, carbohydrate metabolism and amino acid metabolism were significantly increased in the hemolymph of diseased scallop, while the pathway of bacterial motility proteins exhibited higher abundance in the hemolymph of healthy scallop (Fig. 6b). In the adductor muscle, the abundances of nucleotide metabolism and amino acid metabolism pathways were higher in the diseased scallops, while the pathway of bacterial motility proteins was enriched in the healthy scallops (Fig. 6c). For the intestine, the abundances of carbohydrate metabolism, lipid metabolism and amino acid metabolism pathways were significantly higher in the healthy scallops, while the infectious diseases pathway was enriched in the diseased scallops (Fig. 6d).

## Discussion

The frequent outbreaks of bacterial diseases have greatly impaired the production of Yesso scallop in China in recent years. Because the information about the host associated microbiota in healthy and diseased Yesso scallop is limited, the potential pathogens and pathogenesis involved in Yesso scallop’s disease are still not well understood. In the present study, the relationship between the disease and the variation of host associated bacterial communities was explored for the first time, which would provide useful information for further study on the pathogenesis of Yesso scallop’s disease.

Previous studies have hypothesized that the biodiversity of host microbiota determines the likelihood of pathogenic invasion and disease (De Schryver and Vadstein 2014), including positive diversity–invasibility relationship (Wittebolle et al. 2009), negative diversity–invasibility relationship (De Roy et al. 2013), and resident bacterial assemblages (Stecher et al. 2010). In the present study, the bacterial abundance and community structure changed dramatically rather than the α-diversity of the bacterial community between the healthy and diseased Yesso scallop, which was similar to the previous report in shrimp (Xiong et al. 2015). The bacterial loads signified by 16S rRNA gene copy number were significantly increased in the hemolymph, intestine, mantle and adductor muscle of diseased scallop, in comparison to those of healthy scallop. The bacterial community structures changed obviously in the diseased Yesso scallops based on the β-diversity analysis. However, there was no significant difference of the α-diversity indices of Chao1 and Shannon estimates between the healthy and diseased Yesso scallops. The accumulating evidences have indicated that the disruption of the host microbiota could enhance the risk of pathogenic infection in the invertebrates (Ding et al. 2017; Sato et al. 2013; Xiong 2018). These results suggested that the destabilization of the bacterial abundance and community structure in the tissues of diseased Yesso scallop might increase the disease severity.

It is believed that the changes of bacterial community composition are closely linked with the host’s health. In the present study, obvious changes of the bacterial community composition were observed in the tissues between healthy and diseased scallop. The number of the significantly changed species was much higher in adductor muscle and hemolymph than other tissues, which might be the results of the infection of pathogens in adductor muscle and the immune responses in hemolymph. *Planctomycetes* was found to be enriched in the mantle and adductor muscle of healthy scallop but depleted in the diseased host. *Planctomycetes* has been reported to be associated with various eukaryotic organisms including sponges, ascidians, corals, prawns, macrophytes, and even macroalgae (Lage and Bondoso 2014). It was hypothesized that the macroalgae could benefit from the growth factors or antimicrobial molecules produced by *Planctomycetes* (Lage and Bondoso 2014). The enrichment of *Planctomycetes* in mantle and adductor muscle was suspected to be conducive for the health of Yesso scallop. *Vibrio*, *Francisella* and *Photobacterium* were found to dramatically overgrow and dominate in the mantle, adductor muscle and intestine of diseased scallops, respectively, suggesting that they might play an active role in the disease progression. *Vibrio* is well-known to be the main aetiological agents of mollusc diseases (Romalde et al. 2014). *Francisella* is pleomorphic intracellular bacteria, which can cause zoonotic tularemia in humans and many animal species (Pechous et al. 2009). It was observed to dominate in the adductor muscle lesions of Yesso scallop, and considered to be the probable causative agent of the lesions (Kawahara et al. 2018; Meyer et al. 2017). *Photobacterium* was also considered as a bacterial pathogen for many marine animals including fishes, molluscs, crustaceans, and corals (Moi et al. 2017; Thompson et al. 2005). Moreover, the abundances of the above three bacteria in the farming waters were significantly increased during the onset of disease in our unpublished data, which might be one of the causes of Yesso scallop disease. Interestingly, the abundances of facultatively anaerobic *Vibrio* and *Photobacterium* were found to decrease in the hemolymph of diseased scallop, which might be the consequences of the host immune response triggered by invading pathogens and the increased hypoxia owing to the reduction of filtration activity in diseased scallop (Mchenery and Birkbeck 1986). Therefore, the bacterial community exhibited diverse alteration pattern in different tissues of the diseased Yesso scallop, and the lesions and mortality in Yesso scallop probably resulted from the combined effect of these potential pathogens, which might enhance their virulence through the co-infection.

The change of the bacterial composition reflected the functional variation of the bacterial community in the diseased or healthy Yesso scallop. In the mantle of diseased scallop, the abundance of the gene associated with neurodegenerative diseases was significantly increased, suggesting that the altered bacterial community might damage the host’s nervous system. The hemocytes play key roles in the immune responses of the molluscs (Canesi et al. 2002). The glycogen reserves is a fast energy source, which can be mobilized to meet the increased energy demands for immune defense of scallops (Wang et al. 2012). In the present study, the proportion of the carbohydrate metabolism pathway was found to be higher, indicating that the bacteria in the hemolymph of diseased scallop might compete with the host for energy source, and then impaired the energy supply for the hemocytes in the immune processes. Intestinal microbes can benefit the host by improving the nutrients digestion (Clemente et al. 2012). The dysbiosis of the intestinal microbiota is closely associated with the host’s diseases and metabolic syndrome (Mosca et al. 2016; Xiong et al. 2015). In the intestine of diseased Yesso scallop, the abundance of the metabolism associated pathways including carbohydrate metabolism, lipid metabolism and amino acid metabolism were significantly decreased, while the abundance of infectious diseases associated pathway was significantly increased, suggesting that the changed intestinal microbiota might affect the nutrients absorption and cause the disease of Yesso scallop.

In conclusion, all the results above demonstrated that the bacterial communities changed obviously in the tissues of diseased Yesso scallop. The overgrowth of many potential pathogens in different tissues might contribute to the occurrence of Yesso scallop’s disease. The changed bacterial communities might impair the host’s nervous system, energy supply for immune defense and nutrients utilization. The results were valuable for the diagnoses of Yesso scallop’s disease, and provided significant insights into the etiology of the disease in Yesso scallop.

## Supplementary information

**Additional file 1: Table S1.** Quality sequences and OTU number of the samples.

**Additional file 2: Fig. S1.** Rarefaction curves of different samples.

## Data Availability

The data supporting the conclusions are presented in this published article and the additional materials.
