# Supplementary material for: The differences of bacterial communities in the tissues between healthy and diseased Yesso scallop (Patinopecten yessoensis)
Source: AMB Express. 2019 Sep 14;9:148. doi: 10.1186/s13568-019-0870-x (PMC6745042; doi:10.1186/s13568-019-0870-x)
Supplement: Supplementary file 1 — Additional file 1: Table S1. Quality sequences and OTU number of the samples. [file 13568_2019_870_MOESM1_ESM.docx]

**Table S1** Quality sequences and OTU number of the samples.

| Samples | Filtered quality sequences (n=3) | OTU Number (n=3) |
| --- | --- | --- |
| HE_*D* | 40,833, 63,194, 31,405 | 443, 437, 433 |
| HE_*H* | 55,743, 22,953, 64,933 | 418, 401, 427 |
| MA_*D* | 15,967, 24,840, 44,748 | 209, 409, 380 |
| MA_*H* | 31,434, 7,877, 45,200 | 397, 243, 399 |
| MU_*D* | 55,498, 67,053, 56,213 | 426, 395, 257 |
| MU_*H* | 38,725, 30,877, 28,475 | 434, 452, 249 |
| IN_*D* | 32,212, 34,462, 47,960 | 446, 425, 426 |
| IN_*H* | 29,608, 23,990, 17,187 | 410, 415, 390 |
| SW | 53,574, 44,361, 70,032 | 449, 436, 447 |

HE, hemolymph; MA, mantle; MU, adductor muscle; *H*, healthy; *D*, Diseased.
