# Supplementary material for: The differences of bacterial communities in the tissues between healthy and diseased Yesso scallop (Patinopecten yessoensis)
Source: AMB Express. 2019 Sep 14;9:148. doi: 10.1186/s13568-019-0870-x (PMC6745042; doi:10.1186/s13568-019-0870-x)
Supplement: Supplementary file 2 — Additional file 2: Fig. S1. Rarefaction curves of different samples. [file 13568_2019_870_MOESM2_ESM.docx]

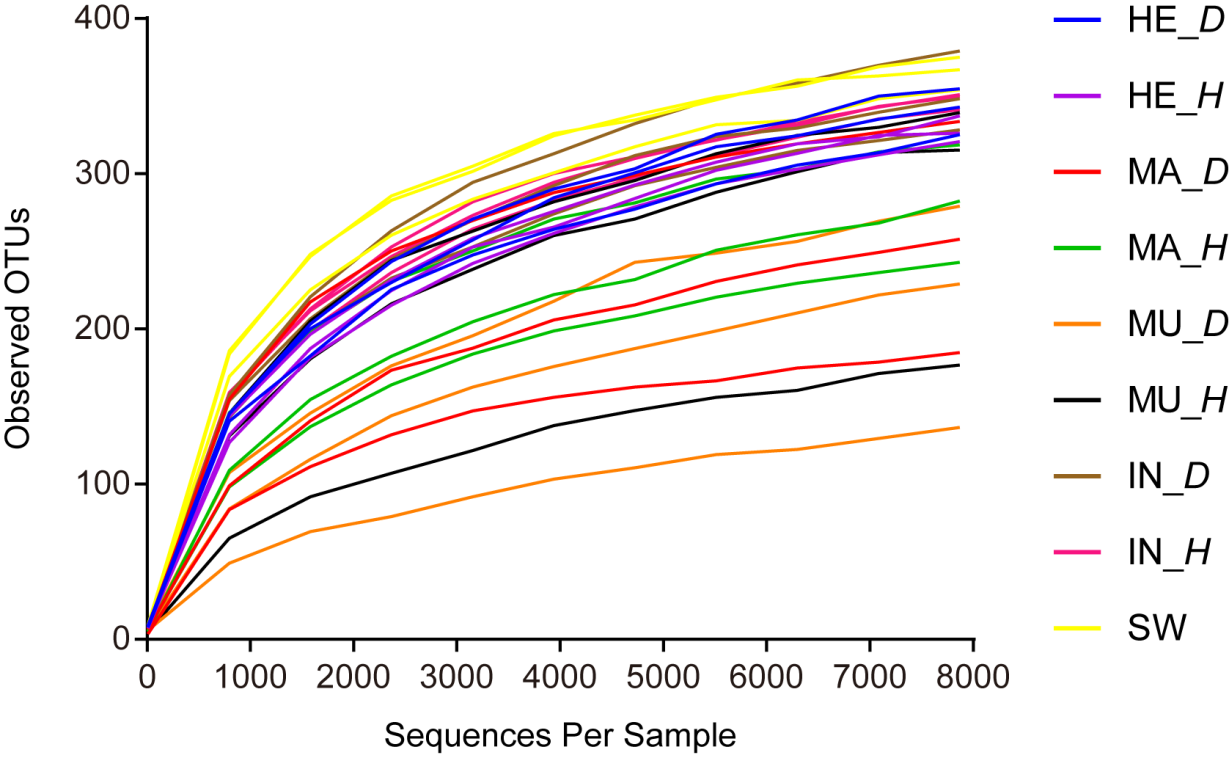


**Figure S1.** Rarefaction curves of different samples. MA, mantle; MU, adductor muscle; HE, hemolymph; IN, intestine; SW, seawater; *H*, healthy; *D*, diseased.
